# A cross-sectional study on serum measles antibody levels of a healthy population in southwest China

**DOI:** 10.1097/MD.0000000000017507

**Published:** 2019-10-25

**Authors:** Lihua Chen, Yuanyuan Xiao, Yahong Yang, Chuanzhi Xu, Shunxiang Li, Tai Zhang

**Affiliations:** a School of Public Health, Kunming Medical University, and Yunnan Center for Disease Control and Prevention, Kunming,; b School of Public Health, Kunming Medical University, Kunming, Yunnan, China; c Department of Chronic Disease Epidemiology, Yale School of Public Health, Yale University, New Haven, CT; d Hospital of Traditional Chinese Medicine in Luliang County, Qujing,; e Yuxi City Center for Disease Control and Prevention, Yuxi,; f School of public health, Dali University, Dali, Yunnan, China.

**Keywords:** antibody level, associated factors, measles

## Abstract

This study aimed to investigate measles antibody level and its associated factors in a healthy Chinese population, so as to provide evidence to measles prevention and control measures in the future.

We conducted a cross-sectional survey by using probability proportionate to size sampling (PPS) among a healthy population aged 8 months to 45 years. Information on measles vaccination status was obtained from the vaccination certificates. Serum measles IgG antibody was detected by enzyme-linked immunosorbent assay. Multivariate logistic and linear regression models were used to analysis the associated factors of measles antibody.

Of the 663 study subjects, the positive rate, protective rate, and geometric mean concentration (GMC) of measles antibodies were 92.76%, 77.53%, 1612.55 mIU/ml, respectively. The antibody concentration was higher in Han ethnical majority than in Hui minority. The positive rate, protective rate and concentration of antibodies in different age groups and regions were clearly disparate. Age, area, and measles-containing vaccine (MCV) immunization history were the prominent influencing factors of antibody positive rate and protective rate. Ethnicity, age, area, and MVC immunization history were the influencing factors of antibody concentration.

Our major findings suggest that, children in rural China, especially in impoverished mountainous regions, were more likely to suffer from delays in measles vaccination. Various measures in optimizing vaccination practices should be implemented in order to prevent possible measles epidemic, even outbreak in these regions.

## 1 Introduction

Measles is a highly contagious disease and one of the major causes of death among children worldwide.^[1]^ The average incubation period of measles is 10 days.^[2]^ The main clinical symptoms are fever, cough, coryza (running nose), red maculopapular rash, Koplik spots, anorexia, diarrhea (especially in infants), and generalized lymphadenopathy. The main complications of measles are otitis media, pneumonia, bronchitis and encephalitis. The risk of death in infant and adult measles cases is higher than in children and adolescents.^[2–3]^ Measles is still a common and often fatal disease in developing countries. Studies have shown that the mortality rate of infant measles cases in developing countries can be as high as 5% to 10%.^[4–5]^ The World Health Organization (WHO) recently announced that there were estimated 89,780 deaths globally from measles in 2016. The overwhelmingly majority (more than 95%) of measles deaths occur in countries with low per capita income and weak health infrastructures.^[6]^

Measles-containing vaccine (MCV) is a live vaccine formed by attenuating measles virus. After an individual is successfully vaccinated, MCV can block the virus from replicating in the nasopharynx, so as to prevent the susceptible person from infecting the virus and avoid spreading the virus to others.^[7–8]^ Before a vaccine was available, measles virus infection was almost universal during the childhood, thus more than 90% of the population attained acquired immunity by the age of 15. Back to then, measles epidemic usually occurred every 2 to 3 years.^[9–10]^ With the use of the vaccines, the incidence of measles has fallen dramatically. In 2000, the United States first announced the elimination of measles nationwide,^[11]^ soon after that, the entire America region achieved the eradication of measles in 2002.^[12]^ The incidence of measles in China has significantly decreased since the introduction of MCV in 1965. Nevertheless, sporadic outbreaks have been reported.^[13]^

Population measles immunization level is the key to determine whether the immune barrier can effectively resist the onset and spread of measles. In order to prevent measles, vaccination is carried out in strict accordance with the specifications of the qualified measles vaccine, and the success rate of immunization can reach about 95%.^[14–15]^ The WHO's strategy to eliminate measles requires the immunity coverage of children is higher than 95%. If the coverage is less than 95%, intensive immunization activities are recommended to prevent possible outbreaks.^[16–17]^ Many reports in China showed that the antibody positive rate is about 90%, and the antibody level in the entire population is lower than which in the aged groups.^[18]^

Ludian county is affiliated to Zhaotong city, which locates in the northeast of Yunnan, a southwest ethnical province of China. In 2014, a magnitude of 6.5 earthquake occurred in Ludian, after that, an emergency vaccination of MCV was carried out for people aged 1 to 14 years. In 2016 to 2017, there were sporadic measles outbreaks and epidemics in Zhaotong.^[13,19]^ In order to learn the level of measles antibody in healthy population in Ludian county after the expanded emergency vaccination, identify susceptible sub-populations, so as to provide evidences for timely and effective vaccination strategies and measures, we carried out this cross-sectional study.

## 2 Materials and methods

### 2.1 Study design

This sampling survey was performed by adopting a multi-stage stratified cluster random sampling method. Before sampling, we divided all townships in Ludian county into 3 strata based on their socioeconomic levels. At the first stage, 2 townships were randomly selected. At the second stage, 3 villages were randomly selected from each chosen township. At the third stage, populations of chosen villages were further divided into 8 age groups: 8 to 17 months, 18 months-2 years, 3 to 6 years, 7 to 14 years, 15 to 24 years, 25 to 34 years, 35 to 44 years, ≥45 years. Based on the sample size calculation formula of simple random sampling (n = 1.5[*z*^2^*P*(1 – *P*) / *e*^2^_]_, *Z* value was 1. 96, and the *e* value was 0.1), the estimated antibody positive rate was set as 0.9 as suggested by existing literature,^[3,13,19,20]^ considering that the sampling error for multi-stage cluster sampling will be bigger, we set a conservative design effect of 1.5 to further adjust sample size. After that, we expanded another 15% of the total sample size to compensate for the possible no response from the participants. The final calculated sample size was 616.

### 2.2 Data collection and management

The survey was implemented between late May and early July in 2018. The participants’ date of birth, ethnicity, sex, and vaccination dates of MCV were extracted from the vaccination certificates. Venous blood (3 ml) was collected from each subject. The serum samples were stored in –20°C environment for further test. Measles immunoglobulin G (IgG) antibody was measured by enzyme linked immunosorbent assay (ELISA) using reagents produced by Zhuhai Haitai Biopharmaceutical Co. Ltd. Criteria: IgG antibody (mIU / ml) <200 was negative, ≥200 was positive, ≥800 was protective.^[22]^

### 2.3 Statistical analysis

Data collected was input into Epidata 3.0, to ensure the quality of inputting, we adopted double-entry strategy. Given the complex sampling design, the survey package in SPSS17.0 was used for all analyses. A 2-tailed *P* value less than .05 was deemed to be significant. *F* test or Chi-squared test was used to compare the GMC, positivity rate and protection rate in different subgroups. Multiple linear regression models were used to analyze the influences of multiple factors, such as age, sex, etc, on concentration. Logistic regression models were used to identify associated factors of positive rate and protective rate.

## 3 Results

### 3.1 Distributive characteristics of population measles antibody level (Table 1).

#### 3.1.1 Sex distribution

We totally surveyed 700 people, 663 of them responded with valid answers, the effective response rate was 94.71%. The positive rates of measles antibody in male and female populations were 90.78% and 94.32% (*χ*^*2*^ = 3.05, *P* = .08); the antibody protective rates were 74.74% and 79.73% (*χ*^*2*^ = 2.33, *P* = .13); the geometric mean concentrations (GMCs) were 1189.52 mIU/ml and 1300.70 mIU/ml (*t* = 1.53, *P* = .13).

**Table 1:**
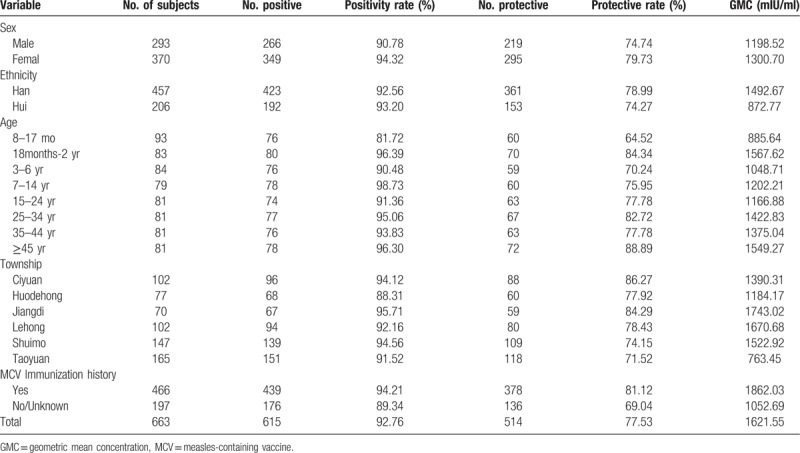
Measles antibody levels in a healthy population in Ludian, 2016–2017.

#### 3.1.2 Ethnic distribution

The antibody positive rates in Han ethnicity and Hui ethnicity were 92.56% and 93.20% (*χ*^*2*^ = 0.09, *P* = .77), and the antibody protective rates were 78.99% and 74.27% (*χ*^*2*^ = 1.82, *P* = .18); the GMCs were 1492.67 mIU/ml and 872.77 mIU/ml (*t’* = 8.86, *P* < .05).

#### 3.1.3 Age distribution

The antibody positive rates in different age groups were 81.72% (8–17 months) and 96.30% (≥45 years) (*χ*^*2*^ = 25.87, *P* < .05), the antibody protective rates were 64.52% (8–17 months) and 88.89% (≥45 years) (*χ*^*2*^ = 21.18, *P* < .05); GMCs ranged between 885.64 mIU/ml and 1549.27 mIU/ml (*F* = 5.098, *P* < .01); pair-wise comparison by using least-significant difference *t* test (LSD-*t* test) revealed expanded differences between chosen age groups.

#### 3.1.4 Space distribution and vaccination history

The antibody positive rates of 6 chosen townships (Ciyuan, Huodehong, Jiangdi, Lehong, Shuimo, and Taoyuan) were 94.12%, 88.31%, 95.71%, 92.16%, 94.54%, and 91.52% (*χ*^*2*^ = 11.04, *P* = .09); GMCs were 1390.31 mIU/ml, 1184.17 mIU/ml, 1743.02 mIU/ml, 1670.68 mIU/ml, 1522.92 mIU/ml, 763.45 mIU/ml (*F* = 16.79, *P* < .05). Pair-wise comparison by using LSD-*t* test found that, the antibody positive rates were all significantly different between chosen townships, except for rates between Huodehong and Taoyuan. The positive rates, protective rates and GMC of measles antibodies in the 2 groups with and without vaccination history were 94.21% and 89.34% (*χ*^*2*^ = 4.88, *P* < .05), 81.12% and 69.04 (*χ*^*2*^ = 11.60, *P* < .05), 1862.03 mIU/ml and 1052.69 mIU/ml (*t’* = 12.893, *P* < .05), respectively.

### 3.2 The associated factors of measles antibody levels

**Figure 1:**
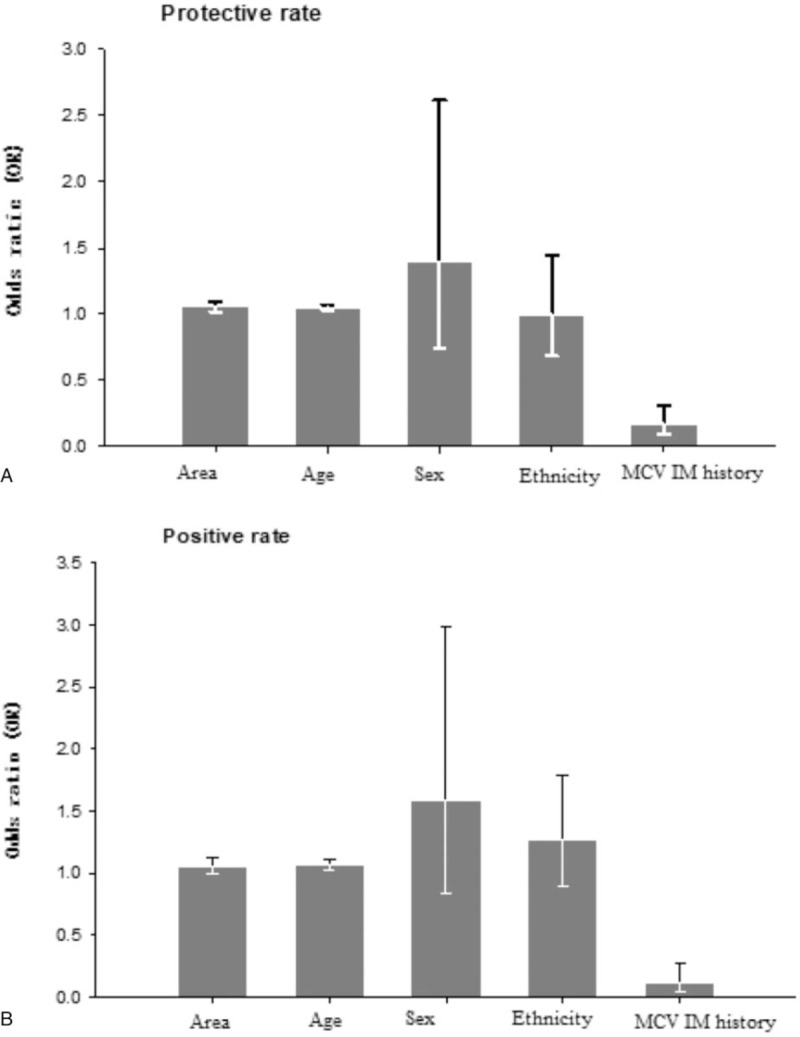
Influencing factors of protective and positive rates by binary logistic regression. In this figure, area (Ciyuan in contrast to Shuimo), age (≥45 years in contrast to 8–17months), sex (male in contrast to female), ethnicity (han in contrast to hui), MCV immunization history (yes in contrast to no).

Binary logistic regression was used to analyze the effects of age, sex, area, ethnic, and vaccination history on protective rate and positive rate. The results showed that the influencing factors of protective rate were area (OR = 1.06, 95% CI = 1.02–1.10), age (OR = 1.05, 95% CI = 1.03–1.07) and MCV immunization history (OR = 0.17, 95% CI = 0.10–0.31). The influencing factors of positive rate were area (OR = 1.06, 95% CI = 1.00–1.13), age (OR = 1.07, 95% CI = 1.03–1.11) and MCV immunization history (OR = 0.11, 95% CI = 0.04–0.28) (Fig. 1).

Multivariate linear regression analysis was used to analyze the effects of age, sex, region, ethnicity, and vaccination history on measles antibody concentration. The results showed that ethnicity, age, area, and MCV immunization history were also the significant influencing factors (Table 2).

**Table 2:**
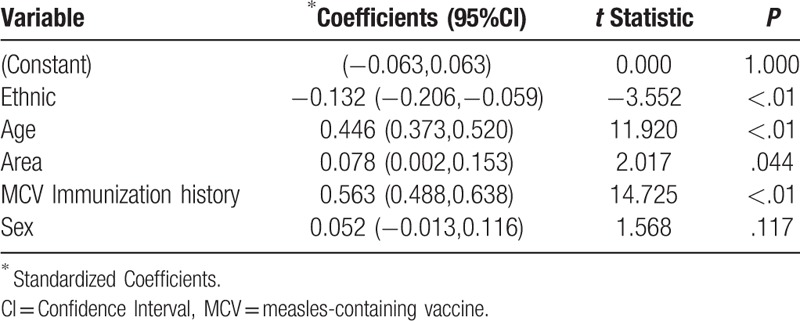
Multiple linear regression analysis of measles antibody concentration in Ludian County.

## 4 Discussion

After the 6.5 magnitude earthquake, emergency vaccination of MCV was carried out for people in Ludian as usually recommended by guidelines. Surveillance results showed that the positive rate of measles antibody level in Ludian county was 92.76%, higher than the antibody level in Xuchang city.^[23]^ The antibody protective rate was 77.53%, higher than which were reported in Honghe^[19]^ and Dali,^[25]^ 2 other counties also locate in the same Yunnan province. When measles antibody concentration was no less than 800 mIU /ml, it can protect against measles virus infection. Nevertheless, when the antibody was between 200 mIU /ml and 800 mIU /ml, although it is tested positive, previous study suggested that a few individuals could contract no rash symptoms of measles.^[24]^ Thus, it was possible that measles cases could occur or epidemic even though the positive rate is high.^[24–25]^ The survey showed that in Ludian county, the concentration of measles antibody level was lower than 800 mIU in Taoyuan township and in age group of 8 to 17 months, so within these 2 subpopulations, the possibility of measles epidemic or even outbreak exists.

The positive rate, protective rate and average concentration of measles antibody in different age groups were significantly different. The positive rate, protection rate and average concentration to 18 months to 2 years old, 7 to 14 years old, 25 to 34 years old, ≥45 years old were relatively high, indicating that these groups of people have good level of measles vaccine immunity. The immunization program for measles vaccination in China started from 8 months old, but the positive rate, protective rate and antibody concentration of 8 to 17 months group were the lowest among all age groups. Many factors may collectively contribute to this low level of immunity. First, based on Chinese immunization program, this age group only vaccinated MCV once, even worse is that, a certain proportion of children within this age group have been found delayed vaccination.^[20]^ Second, previous studies which integrated the protection motivation theory model, health belief model and the theory of planned behaviors found that, children whose guardians had poor knowledge on measles, measles vaccine and immunization schedules were more likely to be vaccinated lately or unvaccinated.^[26,27,28]^ Most guardians of children living in rural are old, poor and less educated, thus they may have poorer knowledge, traditional misconceptions and a weak health belief on timely vaccination.^[29]^ It has been proved that health education is the main prevention and control measure to improve standardized vaccination of MCV.^[20,25]^ Healthcare professionals should receive more support on proper education of immunology, have timely access to up-to-date information on vaccines, and are able to gain technical support from experts in this field. Functional vaccine information system should also be set up and duly maintained to facilitate proper vaccination.^[30]^

The findings of this study revealed that there was a significant difference in the protective rate and GMC of antibodies between different regions in Ludian county. The townships with higher protective rates and GMCs were Ciyuan and Jiangdi, whereas the townships with lower protection rates and GMCs were Lehong and Huodehong. Further, we have provided pair-wise test results between all age groups by using LSD-*t* test found that Huodehong township measles concentration was statistically different from that in other places. We found that the township with a higher measles incidence also had a worse completeness and timeliness of vaccination. In this light, differences in level of completeness and timeliness could be the main reason of measles antibody variation between different regions. Besides, Huodehong township is located in the edge of the mountainous area of Ludian County. Compared with other townships, the traffic transportation is less convenient. Also, there are lots of migrant workers, which could result in a low level of MCV immunization.^[12]^ With the development of the Sino-ASEAN (Association of Southeast Asian Nations) economic zone, more and more parents are migrating from rural to urban areas to find jobs. Such population migration resulted in large numbers of left-behind children in rural hometowns. Previous studies have highlighted that these children were more likely to suffer from health, education, and nutritional problems, due to the absence of parental care.^[31]^ Thus, the regional differences in proportions of parental migration may impact the heterogeneity of vaccination timeliness in this area, which can further cause the variation in measles incidence.^[1]^ In general, it seems that the measles immunization program was unevenly carried out in Ludian county, there were vulnerable populations in the mountainous areas. These findings highlight the necessity to further optimize vaccination policies and measures in rural China so as to reduce inequities in timely vaccination.^[29]^

Although emergency vaccination after the mega earthquake in Ludian generally brought about higher protection rate and antibody concentration of measles antibody, some problems had also been revealed: under-vaccinated or vaccination blank areas still exist. These findings have important secular significance for the prevention and control of measles in ordinary areas where emergency vaccination was not carried out. Regularly assess the level of measles immunization, monitor the level of measles antibody are essential in rural China, as well as other countries with a similar socioeconomic context.

Several limitations of this study should be noticed. First, our research was based on cross-sectional nature, thus causal inference cannot be reached. Secondly, a total of 700 eligible children were surveyed, and the effective response rate was 94.71%, less than the optimum 95% cut-off, thus a small chance of selection bias could exist.

## Author contributions

**Data curation:** Yahong Yang, Shunxiang Li, Tai Zhang.

**Funding acquisition:** Chuanzhi Xu.

**Writing – original draft:** Lihua Chen.

**Writing – review & editing:** Yuanyuan Xiao, Chuanzhi Xu.
